# Insights into Vascular Changes in Hip Degenerative Disorders: An Observational Study

**DOI:** 10.3390/jcm14165845

**Published:** 2025-08-18

**Authors:** Riana Maria Huzum, Bogdan Huzum, Marius Valeriu Hinganu, Ludmila Lozneanu, Fabian Cezar Lupu, Delia Hinganu

**Affiliations:** 1 Department of Radiology, Faculty of Medicine, “Grigore T. Popa” University of Medicine and Pharmacy, 400347 Iasi, Romania; riana-maria.huzum@umfiasi.ro; 2 Department of Orthopedics and Traumatology, Faculty of Medicine, “Grigore T. Popa” University of Medicine and Pharmacy, 400347 Iasi, Romania; bogdan.huzum@umfiasi.ro; 3 Department of Morpho-Functional Sciences I, Faculty of Medicine, “Grigore T. Popa” University of Medicine and Pharmacy, 700115 Iasi, Romania; ludmila.lozneanu@umfiasi.ro (L.L.); hinganu.delia@umfiasi.ro (D.H.); 4 Department of Mechanical, Mechatronics and Robotics Engineering, Mechanical Engineering Faculty, “Gheorghe Asachi” Technical University of Iasi, 700050 Iasi, Romania; fabian-cezar.lupu@academic.tuiasi.ro

**Keywords:** hip joint anatomy, vascular ultrastructural features, microvascular patterns, hip joint immunohistochemistry

## Abstract

**Background**: The epiphyseal vascularization of long bones generates a particular flow pattern that is important for adequate angiogenesis to be achieved. Imaging reveals that vessel development in murine long bone involves the expansion and anastomotic fusion of endothelial buds. Impaired blood flow leads to defective angiogenesis and osteogenesis and downregulation of Notch signaling in endothelial cells. We examined whether altered blood flow and endothelial signaling via the Notch pathway—a highly conserved cell–cell communication mechanism that regulates angiogenesis and vascular remodeling—contributes to hip joint degeneration. **Material and Methods**: In our study, we used two groups of patients. The first is a control group of 15 patients without degenerative joint pathology. The second group consists of 51 patients diagnosed with an advanced form of degenerative joint pathology. On both study groups, we used immunohistochemical markers that highlight the endothelium of epiphyseal capillaries, the collagen matrix, and the presence of joint lubricant-secreting cells. Ultrastructural analysis was performed on hematoxylin-eosin slides that were exposed to a surface electron microscope, following a previously tested protocol. **Results**: The results of our study show that there are numerous anastomoses between epiphyseal vessels and that these capillaries persist even after pathological bone resorption, for a certain period of time. **Discussions:** Our results are complementary to recent studies on this research topic that emphasize the possibility that the main cause of joint degeneration is vascular. Revascularization of an area of bone demineralization after bone infarction has become a reality. **Conclusions**: This study opens new perspectives regarding the research on epiphyseal capillary vascularization and the modern concept of morpho functional rehabilitation of the hip joint.

## 1. Introduction

Degenerative pathology of the hip joint is one of the most common and disabling musculoskeletal conditions, with a significant impact on the quality of life of patients and on the resources of health systems globally. Diseases such as osteoarthritis and avascular necrosis of the femoral head are encountered with increasing frequency among the adult population, especially in the context of increasing life expectancy among the population [1]. They cause decreased mobility, chronic pain, and progressive and irreversible loss of joint functionality. Although numerous studies have explored the etiology and pathophysiology of these diseases, many unknowns remain that are related to the complex situations that determine the onset and progression of joint degeneration. Among the most widely circulated hypotheses regarding the onset of degenerative processes is the involvement of the vascular factor. In particular, the femoral epiphyseal bone and articular cartilage, due to their extremely metabolically active nature and their complex ultrastructure, depend essentially on efficient vascularization [2].

It is believed that the qualitative and quantitative degeneration of this blood flow—either through micro thrombosis or through terminal vascular obstructions—contributes to imbalances between the processes of osteogenesis and bone resorption [3]. These imbalances favor the occurrence of microfractures by reducing bone density and, ultimately, the collapse of the architecture of the femoral head and, implicitly, of the articular cartilage [4].

At the microscopic and ultrastructural level, the epiphyseal bone is composed of networks of fine, biomechanically oriented trabeculae, among which vascular sinusoids are found, which are essential for supporting the vitality of osteocytes and osteoblastic progenitor cells. This fine vascular network is, however, vulnerable to systemic degenerative changes, as well as to local compression or inflammatory factors [5]. In this context, histometric and ultrastructural studies can provide extremely valuable information on the health status of the bone tissue and its vascularization. The assessment of the capillary diameter, vascular wall thickness, microvascular distribution, and relationship between vascularization and the bone matrix provides an integrative perspective on the pathogenesis of degeneration.

The Notch signaling pathway is a highly conserved cell–cell communication system that is involved in the regulation of endothelial cells’ fates, vessel sprouting, and arterial specification. In the skeletal system, Notch signaling also influences the balance between osteoblast and osteoclast activity and thereby plays a crucial role in bone remodeling and microvascular integrity. Disruption of Notch signaling has been implicated in abnormal angiogenesis and osteoarthritis-related changes.

At the same time, the recent literature highlights that vascular changes may precede the appearance of obvious macroscopic or imaging lesions, suggesting that early diagnosis based on histometric and ultrastructural parameters could guide therapeutic interventions before irreversible structural lesions set in. This also gains relevance in light of current studies exploring regenerative or angiogenic therapies that are aimed at restoring local microcirculation and slowing disease progression [6].

Moreover, the comparison of degeneratively affected bone tissue with healthy bone, from a histological and ultrastructural point of view, can reveal not only pathogenetic mechanisms, but also possible individual differences related to age, sex, comorbidities, or lifestyle [7]. From this perspective, a deep understanding of the vascular and architectural changes associated with bone degeneration could significantly contribute to the individualization of treatment and the development of new therapeutic paradigms.

In this scientific framework [8,9], the present study aims to contribute to the deepening of the knowledge regarding the microvascular, histometric, and ultrastructural changes of the femoral head and its associated articular cartilage, in the context of degenerative hip diseases. This observational study was carried out on histological and ultrastructural samples from patients diagnosed with degenerative coxofemoral pathology who were undergoing surgery. The main objectives were to quantify vascular changes through precise histometric parameters, highlight the ultrastructural characteristics of trabecular bone, and analyze the relationships between the vascularization, bone architecture, and clinicopathological data of the patients.

Through this integrative approach, this study aims to provide relevant data for the medical and scientific community, facilitate the identification of histological biomarkers of severity, and open new horizons in the understanding and treatment of degenerative hip diseases.

## 2. Materials and Methods

### 2.1. Material

Our study includes two groups of patients, from whom tissue samples were collected from the femoral head and its articular cartilage. The pieces were collected intraoperatively, following hip joint prosthesis interventions, or from formalized cadavers.

To ensure consistency, all femoral heads were sectioned following a standardized protocol. Each specimen was oriented anatomically, with the femoral head aligned according to the femoral neck axis and the coronal plane defined by the orientation of the fovea capitis and the head–neck junction.

Sections were performed at uniform intervals of 3–4 mm in the coronal plane, starting from the medial margin of the fovea capitis and moving toward the lateral articular surface. Each section was made perpendicular to the axis of the femoral neck using a custom-fabricated cutting jig to ensure anatomical precision and reproducibility. This approach ensured a consistent and comparable orientation across specimens and allowed reliable morphological comparisons.

In our study, all cadaveric specimens were obtained within 24–36 h post-mortem and were promptly fixed in formalin to limit autolysis. Although minor differences in ultrastructural detail were occasionally noted, the overall histological architecture and immunoreactivity—particularly for endothelial markers such as CD31 and ERG—remained comparable across all samples.

We emphasize that these differences did not impact the validity of our findings. All specimens were processed using identical protocols, and our analyses focused on structural and cellular characteristics that remained consistently interpretable in both tissue types.

The first group is a control group consisting of 15 patients without degenerative hip pathology. Of these, 11 were operated on for post-traumatic hip prosthesis and 4 were formalized cadaveric pieces. The latter were collected from male cadavers, aged under 40 years.

Of the 11 patients who were operated on, 5 were female and 6 were male, and all were aged between 27 and 42 years.

The second study group consisted of 51 patients who were diagnosed with an advanced form of degenerative hip joint pathology. They underwent femoral head prosthesis intervention. The age of these patients ranged from 48 to 71 years. A total of 33 were male and 18 were female.

The patients from both groups that were operated on belong to the Orthopedics and Traumatology Clinic of the “Sf. Spiridon” Hospital in Iași. From each patient, tissue fragments were taken from the coxofemoral joint (articular surface of the femoral head and articular cartilage). The tissue samples from this study group were collected during surgical procedures that preserved the integrity of the joint. These patients underwent surgery for surgical neck fractures or anatomical fracture of the femur, as well as for joint prosthesis through the direct lateral Hardinge approach.

The inclusion criteria in these study groups consisted of the recommendation of surgical approach to the hip joint and femoral head prosthesis.

The common exclusion criteria of the subjects in the two study groups were:

- Refusal of surgery;
- Uncooperative patients or relatives;
- Patients or relatives who did not give their consent to participate in this study;
- Patients who could not undergo surgery due to associated pathology;
- Presence of a congenital bone malformation of the spine and/or lower limbs;
- Pathologies associated with the osteoarticular apparatus that can influence posture and can determine vicious positions of the bones of the lower limbs and spine.

The particular exclusion criteria of the first group were:

- Age over 50 years;
- Presence of degenerative pathology of the hip joint;
- Presence of obliterating arteriopathy, in its various clinical forms.
- The exclusion criteria from the second study group were:
- Lack of degenerative pathology of the hip joint, regardless of age;
- Degenerative pathology of the hip joint of a cause other than senescence.

As part of the study design, we established strict exclusion criteria that ruled out any subjects with known risk factors or comorbidities associated with decreased femoral head perfusion. Specifically, cases with a history of corticosteroid use, alcohol abuse, trauma, or previously diagnosed osteonecrosis were excluded. This ensured that all included specimens represented degenerative joint disease (DJD) without overlapping ischemic etiologies.

All patients in the second study group suffered from a cardiovascular disease: 43 were diagnosed with hypertension, 18 with chronic obliterating arteriopathy, 11 with cerebrovascular accidents with minor sequelae, and 16 with coronary artery disease that required vascular prosthesis. The diagnosis of osteoporosis was established using BMD T-scores, which were correlated with imaging findings.

### 2.2. Methods

#### 2.2.1. Histological Study

All histological sections were performed according to the above-described protocol for sectioning the femoral head, at distances between 5 and 15 mm from the fovea capitis. Collagen fibers and endothelial cells were clearly distinguished based on the distribution of interstitial connective tissue fibers. Interstitial connective tissue contains Ki67, CD68, CD31, SOX9, anti-ERG, and anti-lubricin. Ki67 showed an irregular distribution, being abundant in the Haversian system, specifically in its deep part. CD68 was identified in the tissue at the base of the endothelial cells, with a similar distribution in all the studied pieces, while SOX9 was maintained in the connective tissue.

The study specimens were stained using hematoxylin, eosin (HE), and Masson’s trichrome (Van Gieson) (VG) stains. Immunohistochemical staining for Ki67 (1:100 dilution), CD68 (1:300), CD31 (1:100), SOX9 (1:1000), anti-ERG (1:1000), and anti-lubricin (1:250) was assessed on a scale from 0 to 3, according to a histological grading system based on the amount of epithelial fibers and cells, respectively, the amount of interstitial tissue, and the intensity of the staining. Our subjective qualitative assessment was as follows: (0) for negative results; (1) + small amounts/weak intensity; (2) ++ moderate amounts/moderate intensity; (3) +++ large amounts/strong intensity [10,11].

Ki67 showed diffuse nuclear staining throughout the stroma, reproducing the characteristic distribution in proliferative areas. The amount and intensity ranged from moderate to strong. Immunohistochemical analysis of CD31 showed that the antibody was predominantly expressed with moderate to strong intensity on the epithelium lining blood vessels. SOX9 was present in connective tissue.

#### 2.2.2. IHC (Immunohistochemistry) Protocols

Tissue samples were excised for histological and immunohistochemical examination. Human tissue sections were stained with antibodies for Ki67, CD68, CD31, SOX9, anti-ERG and anti-lubricin. Each tissue section included positive controls (normal connective tissue elements, endothelial cells, and interstitial tissue).

Formaldehyde-fixed human tissues were embedded in paraffin wax, then sectioned and incubated in phosphate-buffered saline (PBS), and 4 μm sections were cut for IHC staining. Heat-induced epitope retrieval with citrate buffer (pH 6.0) was performed before peroxide blocking for 10 min. Staining was performed using the following antibodies:

- Ki67, dilution 1:100;
- CD68, dilution 1:300;
- CD31, dilution 1:100;
- SOX9, dilution 1:1000;
- Anti-ERG, dilution 1:1000;
- Anti-lubricin, dilution 1:250.

Sections were developed using a specific HRP/DAB detection kit for mouse and rabbit, with the addition of HRP-conjugated secondary antibodies (biotinylated goat anti-polyvalent secondary antibodies) for 10 min, followed by the addition of streptavidin peroxidase. Counterstaining with hematoxylin was used to visualize the morphology of connective fibers and endothelial or interstitial cells. Images of histological slides were taken at different magnifications using a camera attached to an optical microscope.

Sections harvested from the coxofemoral joint were fixed with formaldehyde and embedded in paraffin wax, then the deparaffinized sections were incubated in phosphate-buffered saline (PBS), and 4 μm sections were made for IHC staining. Heat-induced epitope retrieval with citrate buffer, pH 6.0, was performed before peroxide blocking for 10 min and staining with antibodies Ki67 (clone MM1, Abcam, Cambridge, UK), dilution 1:100, CD68 (clone 514H12, Abcam, UK), dilution 1:300, CD31 (clone 1A10, Abcam, UK), dilution 1:100, anti-ERG [EPR3864] (ab92513, Abcam, UK), dilution 1:1000, anti-SOX9 [EPR14335-78] (ab185966, Abcam, UK), dilution 1:1000, and anti-lubricin/MSF (ab28484, Abcam, Cambridge, UK), dilution 1:250. Sections were developed using the mouse- and rabbit-specific IHC HRP/DAB detection kit with HRP-conjugated secondary antibodies (biotinylated, goat anti-polyvalent) for 10 min and streptavidin peroxidase, and then counterstained with hematoxylin to visualize the morphology of collagen fibers, endothelial cells, or chondrocytes. Images of the histological slides were taken at various magnifications with a camera attached to an optical microscope.

#### 2.2.3. Surface Electron Microscopy (SEM) Study

The ultrastructural study was performed on the same group of patients included in the histological and immunohistochemical studies. Samples collected from the coxofemoral joint of patients who underwent surgical interventions for surgical or anatomical femoral neck fractures, as well as for joint prosthesis, were fixed with formaldehyde and embedded in paraffin wax. Donations from the “Ion Iancu” Institute of Anatomy of our University were also used as samples.

In total, 66 specimens were analyzed using scanning electron microscopy (SEM): one from each patient in the degenerative joint disease (DJD) group and one from each patient in the control group. The selection was based on two main criteria: (1) optimal preservation of trabecular architecture during the initial fixation and decalcification process, and (2) representation of median disease severity (for DJD cases), as assessed by radiographic and macroscopic grading. These criteria were chosen to ensure both technical feasibility and representative comparison between pathological and non-pathological conditions.

SEM is used to analyze metallic, non-metallic, and biological materials through three working modes: high vacuum, low vacuum, and environmental SEM (ESEM). In order to obtain high-quality images, the high-vacuum working mode was used, and the samples were properly prepared by dehydration. An essential element of electron exploration is that the property of the sample surface needs to be conductive, and for this purpose the sections were covered with a superficial layer of gold with a thickness of 7 nm.

A state-of-the-art scanning electron microscope (SEM) VegaTescan LMH II (Tescan, Brno, Czech Republic) was used. The technical features of the microscope are as follows: 100,000× magnification, secondary electron (SE) detector, high vacuum, tungsten filament, 7-sample carousel (standard dimensions 10 × 10 × 45 mm), VegaTescan software, version 35.0.0.

For the detailed analysis of the microvascular architecture and articular surfaces in degenerative hip pathology, SEM was used, with a special technical adaptation applicable to histological sections that are already stained conventionally with hematoxylin-eosin (HE) [11,12]. This approach allowed the valorization of the existing operative biological material, providing a three-dimensional and ultrastructural perspective that was complementary to the classical morphological investigation.

Tissue pieces were initially processed by standard histopathological technique, including fixation in 10% buffered formalin, dehydration in increasing alcohol series, clarification in xylene, and inclusion in paraffin. The obtained sections (4–5 µm thick) were stained with hematoxylin-eosin and mounted on glass slides with coverslips using permanent mounting media. Subsequently, to prepare the samples for SEM analysis, the slides were carefully disassembled by successive treatment with an organic solvent compatible with synthetic resin-based mounting media. This step allowed the recovery of the intact histological section, without affecting the structure of the pre-stained tissues.

After disassembly, the sections were allowed to dry completely in a controlled environment at room temperature, then they were placed on appropriate metal supports (stubs) for SEM. To ensure the conductivity necessary for examination in vacuum, the surface of the samples was covered with an ultrafine layer of gold (gold-sputtering), with a thickness of approximately 5–10 nm, using a sputter coater device in an argon atmosphere. This step was essential to prevent the accumulation of electrostatic charge during electronic analysis.

SEM examination was performed under medium-vacuum conditions, with an accelerating voltage between 5–15 kV, using secondary electron detection modes for surface relief and backscattered electrons for compositional contrast. This method allowed the exploration of fine details of the extracellular matrix, the trabecular bone structure, the vascular organization, and possible changes in mineralization or cartilage degeneration depending on the pathological stage.

#### 2.2.4. Statistical Analyses

From a statistical perspective, the primary objective of this study is to correlate the data obtained through different investigative methods. Specifically, it aims to identify a significant association between the scores derived from scanning electron microscopy (SEM) and those obtained through immunohistochemistry (IHC). The analysis involves evaluating the presence of a positive correlation between the degree of morphological degradation (as assessed by SEM scores) and the intensity of pathological marker expression (as reflected by IHC scores).

To demonstrate this relationship, appropriate statistical tests were applied, including Pearson’s correlation coefficient (for normally distributed data) or Spearman’s rank correlation coefficient (for ordinal data), both of which quantify the strength of association between the two sets of variables. A high coefficient value (e.g., ρ > 0.6) would indicate a direct, statistically significant correlation between structural damage and molecular expression, supporting the hypothesis that tissue deterioration is accompanied by enhanced immunohistochemical reactivity. A significant positive correlation (e.g., ρ > 0.6, *p* < 0.05) would confirm that, as tissue degradation progresses, pathological molecular expression intensifies.

Thus, the statistical findings validate the existence of a consistent relationship between the morphological degree of tissue impairment and its biochemical profile, reinforcing the relevance of both assessment methods in the evaluation of degenerative diseases.

This study was conducted in accordance with the Declaration of Helsinki, and approved by the Institutional Ethics Committee of Grigore T. Popa University of Medicine and Pharmacy, Iasi, Romania, protocol code 284 on 5.03.2023.

## 3. Results

In this study, a comprehensive assessment of the changes occurring in the hip joint in the context of degenerative pathology was conducted, with an emphasis on histological, immunohistochemical, and ultrastructural (SEM) alterations, as well as on the statistical analysis of the obtained data. The investigations targeted both the articular cartilage of the femoral head and the subchondral trabecular bone, aiming to highlight the correlations between the progressive destruction of the cartilage matrix and adjacent bone remodeling. This integrative approach provides a detailed picture of the cellular and tissue mechanisms that contribute to the evolution of joint dysfunction in degenerative diseases of the hip.

### 3.1. Results of Histological Study

**Figure 1:**
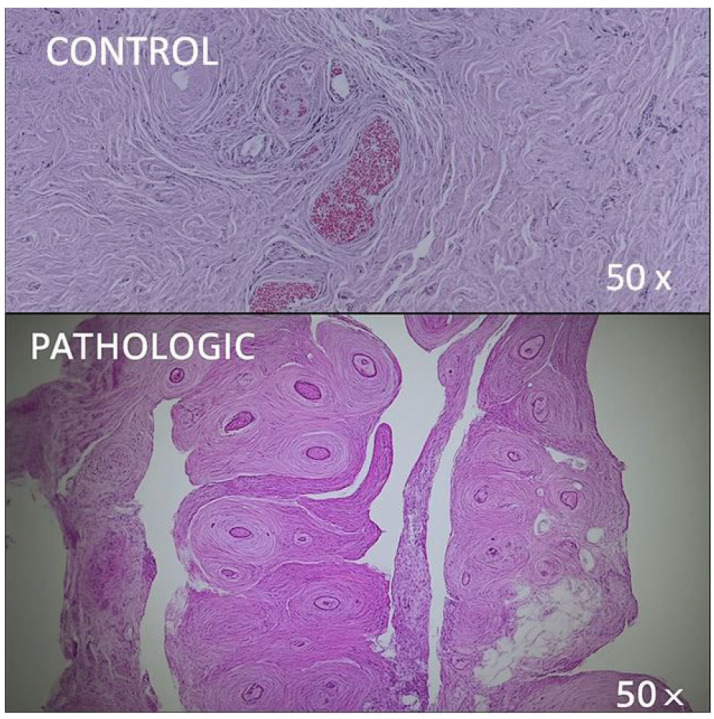
Comparative histological aspect of femoral head trabecular bone, HE stain, 50× magnification. CONTROL: normal trabecular architecture with regular lamellar organization and evenly distributed osteocytes in lacunae. PATHOLOGIC altered trabecular structure with disorganized lamellae, widened bone marrow spaces, and areas of trabecular thinning, consistent with advanced degenerative joint disease. Coronal plane, medial-to-lateral sequence, at 7 mm from fovea capitis.

The histological images demonstrate advanced osteoarthritic changes, including chondrocyte hypertrophy, nuclear pyknosis, irregular subchondral trabeculae with disrupted lamellar organization, empty osteocytic lacunae, and early medullary fibrosis. Notably, the articular cartilage exhibits loss of zonal organization, with deep vertical fissures, matrix disintegration, and the presence of chondrocyte clusters in isogenic clones (highlighted in Figure 1).

These findings are characteristic of late-stage degeneration and are further supported by the presence of cartilage fibrillation, trabecular rarefaction, and the altered expression of immunohistochemical markers involved in cartilage and bone metabolism. A statistically significant association was observed between the lesion severity and morphological profiles, which underlines the diagnostic relevance of the histological assessment.

Histological examination of the vascular component of the hip joint highlights the retinacular arteries as the principal source of femoral head perfusion. However, visualization of the intramedullary vasculature was challenging due to the dense, calcified structure of the femoral head. Normal lamellar bone trabeculae were observed, delineating medullary spaces populated with hematopoietic elements and adipocytes. Within these trabeculae, Haversian canals with concentric lamellae and viable osteocytes in lacunae were present, bordered partially by a cement line. This image confirms the preserved architecture of healthy trabecular bone, with no signs of pathological remodeling (Figure 1, CONTROL).

**Figure 2:**
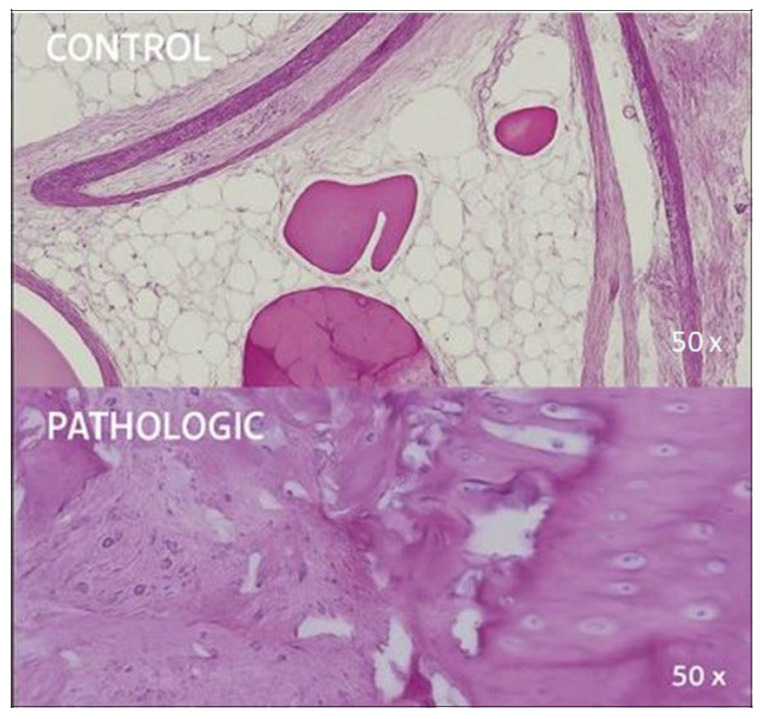
Histological appearance of epiphyseal cartilage and adjacent adipose tissue, HE stain, 50× magnification. CONTROL: intact hyaline cartilage with smooth surface and well-defined chondrocytes embedded in a homogeneous extracellular matrix; adjacent mature adipocytes show regular morphology. PATHOLOGIC: markedly disorganized cartilage structure, with clustered hypertrophic chondrocytes, disrupted matrix continuity, and irregular cartilage–bone transition, suggestive of degenerative remodeling processes. Coronal plane, medial-to-lateral sequence, at 9 mm from fovea capitis.

Regular staining clearly distinguished areas affected by degenerative joint pathology from unaffected regions. Destructive changes in both the articular cartilage and epiphyseal bone are readily visible (Figure 1 and Figure 2).

Further microscopic evaluation reveals hypertrophic chondrocytes with pyknotic nuclei, which indicates cellular degeneration. The subchondral bone exhibits thick, irregular trabeculae, a disorganized lamellar structure, and partially emptied osteocytic lacunae. Narrowed medullary spaces with early fibrotic transformation are also evident. These features are consistent with advanced osteoarthritic transformation.

The epiphyseal trabecular bone shows significant structural and cellular changes. The trabeculae appear variably thickened or thinned, presenting a sclerotic or porotic pattern, respectively, depending on the disease stage. The loss of lamellar alignment, the presence of microfractures, and the observed areas of osteonecrosis (highlighted in Figure 2, PATHOLOGIC) support ongoing degenerative remodeling. Osteocyte lacunae are frequently empty or contain pyknotic cells, which indicates compromised cellular viability. Medullary spaces may show fibrosis, reduced hematopoietic cellularity, and mild lymphoplasmacytic infiltration, especially in advanced cases.

In articular cartilage, the degenerative process is evidenced by surface fissuring, loss of matrix homogeneity, and disruption of zonal stratification. Chondrocytes display nuclear atypia, hypertrophy, and clonal aggregation in dilated lacunae. The matrix shows variable staining intensity, which suggests altered glycosaminoglycan content. In late-stage degeneration, the subchondral bone becomes exposed, which is often associated with marginal osteophyte formation and bone remodeling (Figure 2, PATHOLOGIC).

### 3.2. Results of IHC Study

In order to deepen our understanding of the cellular and molecular mechanisms involved in the degenerative pathology of the hip joint, a comparative immunohistochemical (IHC) study was conducted on two distinct groups of subjects. The aim of this analysis was to evaluate the expression of specific cellular markers associated with proliferation, inflammation, angiogenesis, chondrogenic phenotype maintenance, and cartilage integrity.

The IHC analysis was performed on paraffin-embedded femoral head sections obtained under standardized conditions. Comparing the marker expression between the control and pathological groups enabled the identification of significant differences in proliferative activity, local inflammation, neovascularization, and structural degradation, and thus provided valuable insight into the underlying degenerative mechanisms of hip osteoarthritis.

The marker selection was based on the need for a multidimensional evaluation of joint degeneration that encompassed the proliferative potential (Ki67), inflammatory status (CD68), angiogenesis (CD31, ERG), chondrocyte viability (SOX9), and extracellular matrix integrity (Lubricin/PRG4). This comprehensive panel not only delineates morphological changes but also reveals key biological processes involved in disease progression.

**Figure 3:**
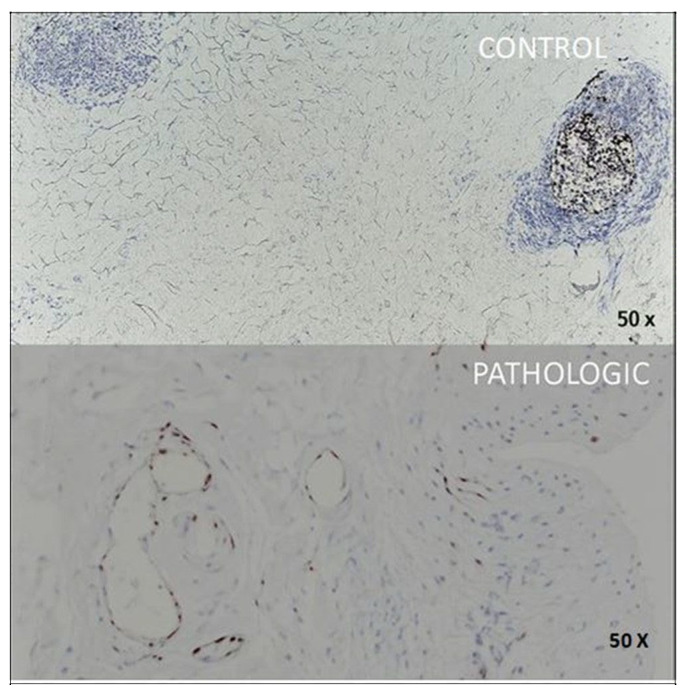
Immunohistochemical expression of Ki67 in femoral head tissue samples, 50× magnification. CONTROL: sparse Ki67 expression confined to scattered nuclei within Haversian systems, surrounded by morphologically normal trabecular bone. PATHOLOGIC: limited Ki67 nuclear positivity (brown) predominantly localized in perivascular areas and endothelial cells of degenerated articular cartilage, indicates reduced proliferative activity in osteoarthritic bone. Coronal plane, medial-to-lateral sequence, at 15 mm from fovea capitis.

For Ki67, the overall hypocellularity and lack of chondrocyte staining reflect its reduced regenerative capacity in advanced osteoarthritic cartilage. This marker, indicative of cellular proliferation, showed localized expression in the Haversian system in control samples, while in pathological samples, its expression was restricted to perivascular endothelial cells, which indicates the diminished regenerative signaling (Figure 3).

A low focal Ki67 nuclear expression is observed in endothelial and perivascular cells, with absent chondrocyte reactivity, which indicates reduced proliferative activity that is typical of advanced osteoarthritic cartilage.

CD68 reveals sparse positive cells that are localized mainly in perivascular and subchondral areas, which indicates limited macrophage infiltration, which is associated with chronic inflammation in advanced osteoarthritic cartilage.

CD68, a specific marker for macrophages, was introduced to characterize the local inflammatory infiltrate. The presence of macrophages in the medullary space and in the vicinity of the subchondral area was correlated with the secretion of proinflammatory cytokines and the mediation of extracellular matrix degradation.

**Figure 4:**
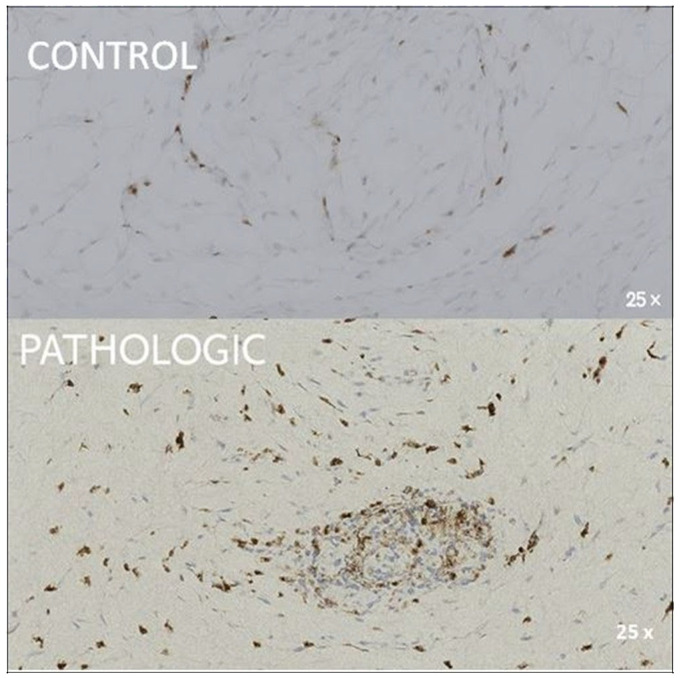
Immunohistochemical expression of CD68 in femoral head tissue, 25× magnification. CONTROL: moderate-to-strong CD68 positivity predominantly localized around Haversian systems, with moderate expression also observed intra-Haversian, reflecting physiologic macrophage distribution. PATHOLOGIC: sparse CD68-positive cells concentrated in perivascular and endothelial regions, with reduced immunoreactivity in cartilage areas. The marked hypocellularity and absence of chondrocyte staining suggest impaired macrophage-mediated remodeling in advanced osteoarthritis. Coronal plane, medial-to-lateral sequence, at 12 mm from fovea capitis.

The macrophage-specific marker CD68 revealed limited inflammatory infiltration in pathological cartilage, with sparse positivity in perivascular and subchondral zones. In control samples, moderate-to-strong expression was observed around the Haversian system, which suggests baseline macrophage activity (Figure 4).

To assess neovascularization—a well-documented pathological process in joint degeneration—two complementary markers were used: CD31 and ERG. CD31, also known as PECAM-1, is a classic marker for vascular endothelium, but has relatively low specificity in inflammatory conditions or in tissues that are rich in hematopoietic cells [5]. For this reason, it was combined with anti-ERG, a nuclear marker that is specific for endothelial cells, which allows a clearer differentiation of pathological micro vascularization and the angiogenic response in trabecular bone and deep cartilage.

**Figure 5:**
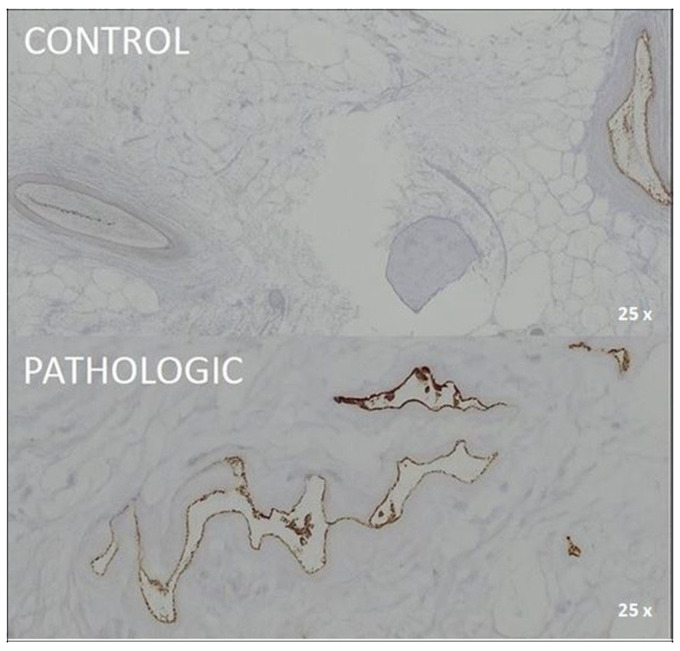
Immunohistochemical expression of CD31 in femoral head bone tissue, 25× magnification. CONTROL: CD31 highlights normal endothelial lining of well-organized trabecular vessels with uniform morphology and distinct lumina. PATHOLOGIC: discontinuous and confluent CD31-positive staining in lacunar or infarcted vessels, indicating vascular rarefaction and structural compromise in the context of advanced osteoarticular degeneration. Coronal plane, medial-to-lateral sequence, at 10 mm from fovea capitis.

**Figure 6:**
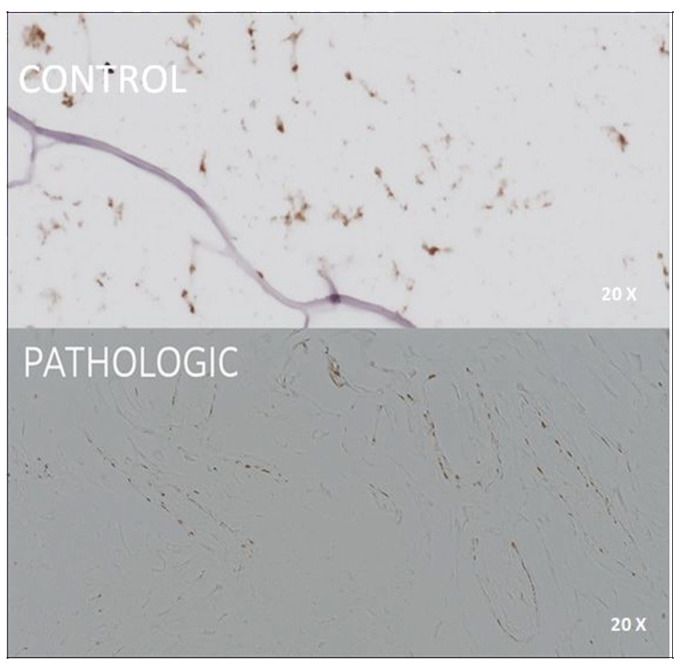
Immunohistochemical expression of ERG in femoral head cartilage, 20× magnification. CONTROL: ERG nuclear positivity (brown) is confined to endothelial cells of subchondral micro vessels, indicating preserved vascular integrity and absence of aberrant angiogenesis. Chondrocytes are ERG-negative, consistent with their non-endothelial identity. PATHOLOGIC: focal and intense ERG nuclear staining is evident in endothelial cells of proliferating vessels, primarily located in the deep cartilage layer and perichondral connective tissue, reflecting localized angiogenic remodeling associated with degeneration. Coronal plane, medial-to-lateral sequence, at 10 mm from fovea capitis.

Two vascular markers, CD31 and ERG, provided complementary insights into the angiogenic response and vascular integrity. CD31 marked normal endothelial cells in control trabecular vessels, while pathological samples exhibited confluent and disrupted patterns that were consistent with vascular rarefaction (Figure 5). ERG, a nuclear endothelial marker, confirmed the presence of angiogenic remodeling in degenerated cartilage (Figure 6).

CD31 marks lacunar, infarcted vessels that exhibit a confluent pattern, which is indicative of vascular compromise

The CD31 marker shows us the vascular endothelium; thus, it highlights vascular lacunae in the case of infarcted bone (Figure 5, PATHOLOGIC) and, at the same time, demarcates the functional trabecular epiphyseal vessels (Figure 5, CONTROL). In both cases, this marker has a moderate-to-strong expression in the epiphyseal bone.

To assess the maintenance of the chondrogenic phenotype, SOX9, a transcription factor that is critical for chondrocyte differentiation and predominantly expressed in healthy chondrocytes in articular cartilage, was used. Decreased SOX9 expression is considered an early marker of chondrocyte dysfunction and cartilage degradation in osteoarthritis.

Anti-lubricin (or PRG4) was selected for its ability to reflect the integrity of the superficial layer of articular cartilage. Lubricin is a glycoprotein that is essential for the lubrication of articular surfaces and the prevention of inter cartilaginous adhesion.

Heterogeneous SOX9 expression in isolated chondrocytes indicates disrupted homeostasis and chondrogenic decline in arthritic cartilage.

**Figure 7:**
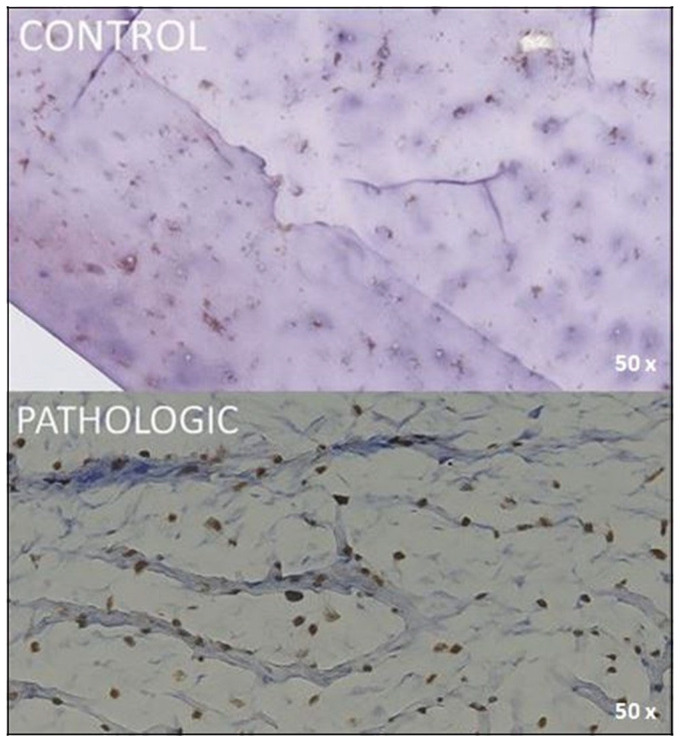
Immunohistochemical expression of SOX9 in articular cartilage and adjacent bone tissue from the femoral head, 50× magnification. CONTROL: nuclear SOX9 positivity (brown) is evident in mature chondrocytes within a well-organized extracellular matrix, indicating sustained chondrogenic transcriptional activity and phenotypic stability. PATHOLOGIC: SOX9-positive nuclei remain detectable in scattered chondrocytes, although expression is diminished. The surrounding matrix is weakly stained, with irregular fibrillar structure and disrupted architecture, suggesting collagen degradation and progressive loss of cartilage integrity. Coronal plane, medial-to-lateral sequence, at 10 mm from fovea capitis.

Positive nuclear reactivity for SOX9 is observed in the majority of chondrocytes, as highlighted by intense brown staining, and indicates normal transcriptional activity associated with functional status and active chondrogenic differentiation. The cartilaginous matrix appears uniform, with well-defined chondrocyte lacunae and no signs of structural disruption, which is consistent with physiologic cartilage or only minimal degenerative changes. No areas of necrosis, inflammatory infiltrate, or major pathological activity are identified (Figure 7, CONTROL). In contrast, scattered and heterogeneous expression in the pathological samples reflected disrupted chondrocyte function and extracellular matrix degradation (Figure 7 PATHOLOGIC).

The presence of isolated and dispersed chondrocytes, with heterogeneous SOX9 expression, reflects the loss of tissue homeostasis and the decline in the chondrogenic phenotype in the context of arthritic lesions (Figure 7, PATHOLOGIC).

Focal nuclear ERG expression is observed in endothelial cells of deep cartilage and perichondral vessels, indicating a preserved vascular identity in pathological articular tissue.

This specific nuclear endothelial marker allows clear highlighting of the remaining microvasculature, even in areas with advanced structural alteration. The image suggests the persistence of a functional vascular network in the degenerated cartilage, which has potential significance in tissue regeneration or lesion progression (Figure 7, PATHOLOGIC).

The weak or absent expression of lubricin was observed in the superficial layer of the cartilage, where this glycoprotein is normally intensely expressed to ensure the lubrication of the articular surfaces and protection against friction.

**Figure 8:**
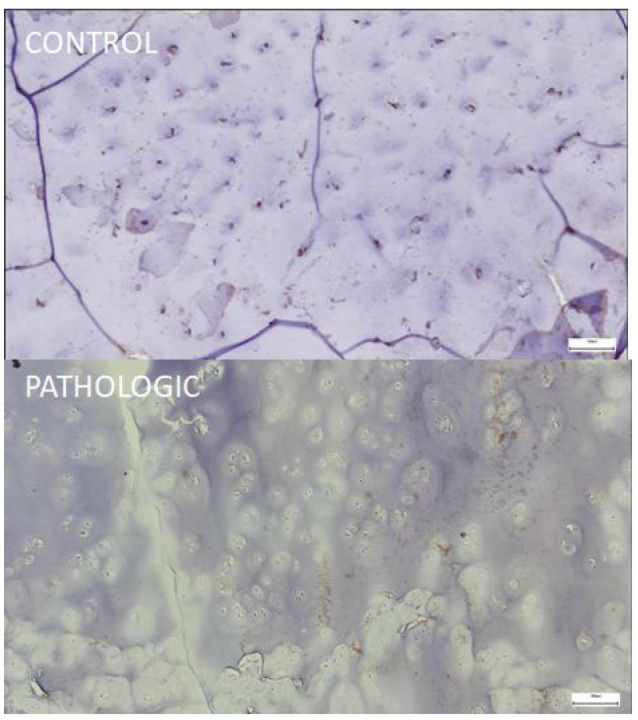
Immunohistochemical expression of lubricin (PRG4) in femoral head articular cartilage, 100× magnification. CONTROL: strong membranous and cytoplasmic PRG4 expression is evident in superficial zone chondrocytes and the adjacent extracellular matrix, supporting effective boundary lubrication and structural preservation of the cartilage surface. PATHOLOGIC: reduced and discontinuous PRG4 immunoreactivity, with focal loss of expression in the superficial cartilage layer, suggests impaired lubricating capacity and early surface fibrillation in degenerative joint disease. Coronal plane, medial-to-lateral sequence, at 12 mm from fovea capitis.

The disappearance of the positive immune signal reflects the loss of functional integrity of the superficial layer, which is characteristic of advanced stages of osteoarthritis. The appearance of the superficial layer is suggestive of progressive degradation of the extracellular matrix and alteration of the cartilage’s ability to maintain joint homeostasis (Figure 8, PATHOLOGIC).

By using this panel of markers, the proposed study provides a detailed picture of the pathobiological processes that contribute to the gradual destruction of the hip joint.

It should be noted that it was not possible to perform immunohistochemical staining on trabecular bone sections, as the standard histological preparation process, which involves demineralization of bone tissue, causes loss of antigenicity and alteration of the structure of epitopes that are required for specific antibody binding. Thus, the immunohistochemical analysis was limited to the soft components of the joint, especially the articular cartilage and perivascular connective tissues.

Together, the findings from this IHC study provide a nuanced view of how the degenerative cascade affects the hip joint, from altered proliferative dynamics and immune infiltration to compromised vascular and chondrogenic function. Notably, antigen preservation challenges limited the analysis of mineralized trabecular bone, reinforcing the focus on cartilage and perivascular soft tissue.

### 3.3. Results of SEM Study

Bone trabeculae exhibit structural disorganization, thinning, fragmentation, and hyper porosity within their lacunar resorption areas, which is indicative of active bone remodeling, osteoclastic resorption, and reduced mineral density that is consistent with osteoporotic changes. SEM imaging at 20,000× reveals ultrastructural cartilage degeneration characterized by collagen network disorganization, matrix porosity, chondrocyte loss, and absence of periendothelial coverage in vessels post-resorption, which is indicative of advanced osteoarthritic damage.

Ultrastructural analysis by SEM allowed for the detailed characterization of morphological changes that occur in trabecular bone and articular cartilage in the context of degenerative hip joint pathology. Compared with the control group, in the pathologic group, the SEM images revealed significant alterations in trabecular architecture, capillary network continuity, and extracellular matrix organization. This method provided a valuable three-dimensional perspective on the processes of demineralization, microfracture, trabecular collapse, and articular surface degradation, supporting previous histological and immunohistochemical data. The SEM results reinforce the hypothesis of a close interdependence between subchondral bone degradation and cartilage destruction in the evolution of degenerative hip diseases.

**Figure 9:**
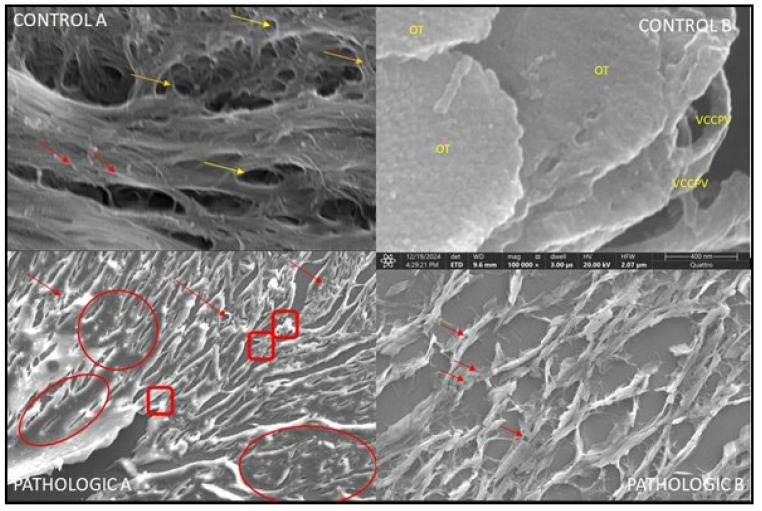
Scanning electron microscopy (SEM) analysis of trabecular bone from the proximal femoral epiphysis in control and osteoarthritic samples. CONTROL A: SEM image at 20,000× magnification showing a well-organized trabecular network with porous, interconnected morphology characteristic of healthy cancellous bone. Red arrows indicate intact capillary endothelium; yellow arrows highlight Haversian canals; brown arrows identify Volkmann canals. CONTROL B: high-magnification image (100,000×) illustrating a normal osteocyte territory (OT) and preserved vascular channels. Capillary vessels with perivascular cells (VCCPVs) are evident, confirming functional bone microcirculation. PATHOLOGIC A: SEM image at 500× revealing areas of bone infarction (red circles), trabecular delamination, and microfractures. Red arrows indicate disrupted or infarcted Haversian capillaries, while red squares highlight perivascular cells in demineralized regions, suggestive of chronic ischemic remodeling. PATHOLOGIC B: image at 2000× magnification showing severe trabecular degradation, with collapsed architecture and prominent infarcted capillaries (red arrows) in areas of advanced demineralization.

In surface electron microscopy images, the ultrastructural appearance of normal epiphyseal bone shows the following features:

Trabecular bone structure:

- The trabecular bone’s appearance, with well-organized trabeculae, is strategically distributed to support mechanical loads;
- The spaces between the trabeculae contain bone marrow or blood vessels.
- Vascular network:
- Small blood vessels (capillaries) that run through the bone, evenly distributed in the Haversian and Volkmann canals (Figure 9, CONTROL);
- The ultrastructural details of the capillary wall show a thin endothelium with mitochondria and elongated nuclei.
- Possible perivascular cells associated with maintaining vascular and metabolic function.

**Figure 10:**
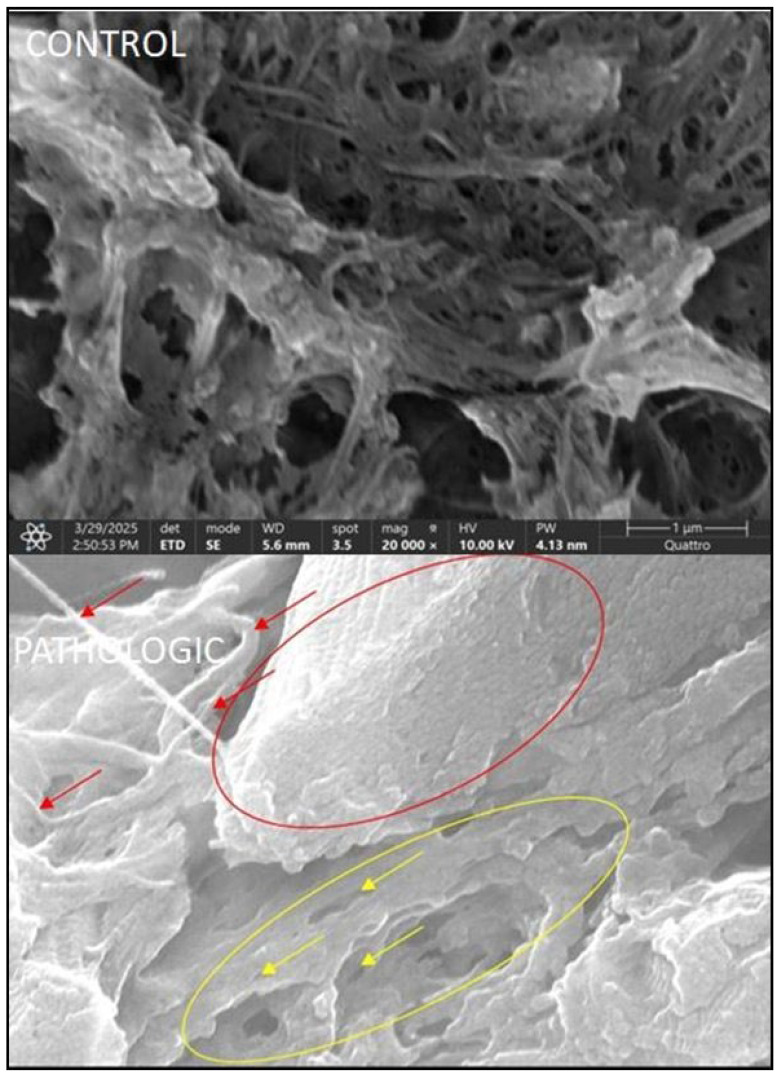
Scanning electron microscopy (SEM) of femoral head articular cartilage and subchondral bone, illustrating differences between control and osteoarthritic samples. CONTROL: SEM image at 20,000× magnification showing the superficial (tangential) zone of hyaline cartilage. The surface is relatively smooth, with fine collagen fibers oriented parallel to the surface, forming a dense, organized network consistent with type II collagen architecture. No visible fissures, vertical clefts, or signs of delamination are observed, indicating preserved structural integrity and absence of degenerative changes. PATHOLOGIC: SEM image at 5000× magnification showing disrupted vascular architecture within epiphyseal bone. Multiple anastomotic vessels (red arrows and circle) are observed in viable regions, while infarcted areas (yellow arrows and circle) display obliterated capillaries and evidence of demineralization, indicating compromised microcirculation and early ischemic remodeling.

Adjacent connective tissue:

- The collagen fibers and extracellular matrix provide support for blood vessels;
- Osteoprogenitor cells are located near the capillaries;
- The fineness of normal vascularization is highlighted, which emphasizes the intimate relationship between bone and the vascular system, which is essential for bone nutrition and regeneration (Figure 10, control).

The trabeculae appear thin, continuous, and without obvious discontinuities, which suggests healthy and well-organized trabecular bone. The surface of the trabeculae is relatively smooth, with small natural irregularities, but without obvious signs of erosion or active resorption. No resorption lacunae (How ship) are observed, which indicates the absence of increased osteoclastic activity. Large, regular spaces corresponding to bone marrow are observed between the trabeculae—these spaces allow vascularization and support hematopoietic function. At this magnification and with this method (SEM), the actual bone cells (osteocytes, osteoblasts, osteoclasts) are not visible, as SEM highlights the surface topography, not the intracellular structure.

In electron microscopy, the resolution allows for the observation of molecular-level interactions, such as cell connections and the detailed structure of the endothelium.

The bone plates we examined show visible lesional features caused by fractures or bone loss. There is a specific ultrastructural difference between the SEM aspects of the two distinct situations—normal bone structure (Figure 9, CONTROLA and B) and capillaries (Figure 10, CONTROL), respectively—in terms of their pathologic features (Figure 9 and Figure 10, PATHOLOGIC A and B).

**Figure 11:**
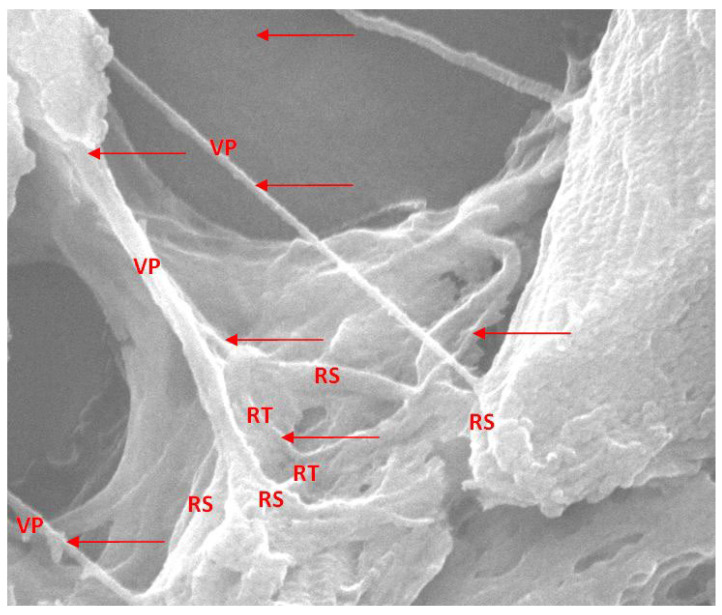
Normal aspects of the microscopic Haversian network and the anastomoses between them (red arrows); fractured bone piece, without bone demineralization. VP = main vessel; RS = secondary branches; RT = tertiary branches; ×3000.

**Figure 12:**
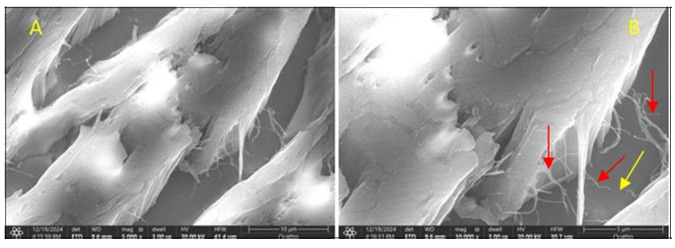
(**A**) = collagen matrix remaining after bone demineralization, seen at a magnification of ×5000; (**B**) = ×10,000; trabecular vascular micro network—red arrows; infarcted capillary vessel—yellow arrow.

By increasing the magnification power, we obtain an image of the collagen matrix left behind by bone demineralization processes (Figure 11). At the same time, we can highlight the bone trabecular vascularization (Figure 12A) in a section of the normal part of the same pathological bone.

Bone demineralization gives us the chance to observe a complex three-dimensional network of trabecular capillary vessels. The blood vessels have not disappeared (have not been resorbed) even after a long period of time following the moment of bone infarction (Figure 12).

The complete bone structure in Figure 11 allows us to objectify only the capillary vessels in the proximity of the structural unit. Unlike the infarcted vascular network in Figure 12, these present a true perivascular cell layer. The latter have completely disappeared in the capillary vessels distal to the bone infarction area.

The main difference is that, in the case of the blood vessels that remain after bone resorption, they are no longer covered by periendothelial cells (pericytes).

An SEM image, obtained at 20,000× magnification, of articular cartilage at the level of the femoral head, in the context of a degenerative lesion, highlights multiple ultrastructural alterations that suggest chronic and irreversible pathological processes. Normally, hyaline articular cartilage presents an organized network of collagen fibers (predominantly type II collagen) that is integrated into an amorphous matrix that is rich in proteoglycans, which supports the biomechanical properties of the joint.

Changes that are observable in an SEM image of degenerative pathology include cracks and erosions of the cartilaginous surface that replace the smooth and homogeneous surfaces of normal cartilage. These mark the beginning of the process of disorganization of the collagen network. Cracks can appear in a network or radial pattern, indicating areas of increased mechanical stress.

Fragmentation and decomposition of collagen fibers appear as discontinuous, disorganized filaments with chaotic orientation and, in advanced stages, these fibers appear fragmented, with areas of discontinuity reflecting the loss of structural integrity.

The reduction in the density of the extracellular matrix is evidenced by its more porous appearance, a sign of proteoglycan degradation and loss of water retention capacity. All these changes are correlated with a decrease in elasticity and an increase in cartilage fragility.

**Figure 13:**
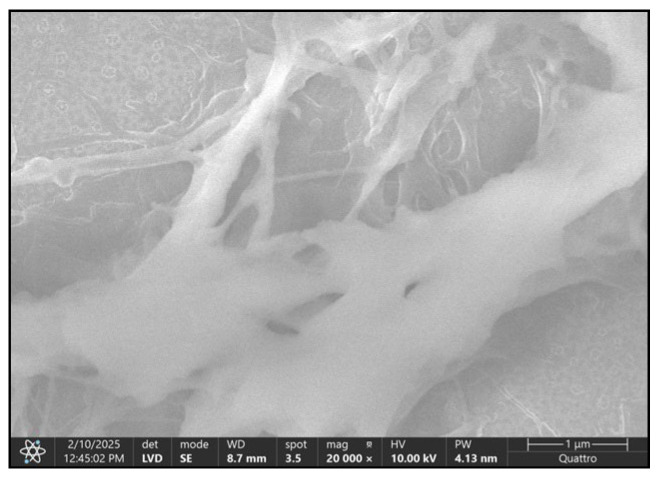
SEM image, obtained at a magnification of 20,000×, of the articular cartilage at the level of the femoral head, in the context of a degenerative lesion, showing multiple ultrastructural alterations suggesting chronic and irreversible pathological processes; deep, irregular cracks (superficial fibrillization) that mark the beginning of the collagen network disorganization process and indicate areas of increased mechanical stress; collagen fiber breakage, which appears in the form of discontinuous, disorganized filaments with chaotic orientation; the matrix having become more porous, a sign of proteoglycan degradation and loss of water retention capacity; chondrocytes visible as empty or deformed lacunae (chondrocyte apoptosis or necrosis).

At this resolution, chondrocytes can be visible as empty or deformed gaps, which is a sign of chondrocyte apoptosis or necrosis and a typical appearance in advanced osteoarthritis. In their place, irregular depressions or collapse of the matrix network can be observed (Figure 13).

### 3.4. Results of Statistical Study

To perform the statistical study, we performed a scoring of the IHC staining intensity on all markers used, as well as one for SEM. We subsequently correlated the statistical results obtained for the IHC archers with those of the SEM image analysis, for each patient. The following tables include immunohistochemical markers as well as SEM images scorings. The IHC images intensity is scored on a standard scale (intensity score 0–3: 0—absent reaction (negative), 1—weak reaction, 2—moderate reaction, 3—intense reaction (Table 1)).

The SEM scores are evaluated on three distinct morphological criteria:

1. Continuity/structural integrity (0–3 points): 0—intact structure, 3—deeply affected structure;
2. Collagen presence/fibrillar organization (0–3 points): 0—collagen absent, 3—well-organized collagen;
3. Vascularization/visible capillaries (0–3 points): 0—complete absence, 3—extensive vascular network.

The total score for each analyzed structure is the sum of the three components, with the maximum being 9 points (Table 2 and Table 3).

#### 3.4.1. Results of Statistical Study of Control Group

The SEM results for the control group, consisting of 15 patients, indicated a score of 0 for all three criteria that were analyzed: structural continuity and integrity, presence of collagen and fibrillar organization, and vascularization and presence of visible capillaries.

Statistically, the scores obtained by the control group for all three criteria analyzed by SEM—structural continuity, collagen fibrillar organization, and vascularization—were constant, with a mean value of 0 and a standard deviation of 0, which indicates perfect homogeneity within this group. This complete lack of variation confirms that the examined tissues (trabecular bone and articular cartilage) do not show signs of structural or biochemical degradation. The presence of well-organized collagen and the absence of vascularization, normal characteristics of healthy cartilage, support that the control group represents a normal morphological standard, without pathological changes that are detectable by scanning electron microscopy.

The IHC scores for the control group range between 0 and 1, indicating, in most cases, the absence of a reaction or a weak reaction to the analyzed immunohistochemical markers and thus reflecting the lack of increased expression of proteins involved in cartilage degradation, bone tissue, or inflammatory processes. The structural and biochemical integrity of the tissues is completely preserved, with no evidence of degeneration, inflammation, or tissue stress. The results obtained by SEM and IHC analysis are perfectly consistent, confirming the joint health of this group. Thus, the control group constitutes an essential benchmark, providing a solid basis for comparison for the evaluation of the changes observed in the pathological group.

#### 3.4.2. Results of Statistical Study of Patient’s Group

From a statistical perspective, the control group serves as a stable standard, without morphological variations, while the pathological group presents significant structural alterations that are quantifiable through high SEM scores. These differences validate the use of SEM scoring as a relevant diagnostic tool, demonstrating that the degradation of bone and cartilage tissue in degenerative diseases is profound and measurable, with clear clinical implications.

The morphological analysis performed using scanning electron microscopy (SEM) highlights significant differences between the control group and the pathological group, in terms of structural damage to the tissues that were analyzed.

The control group presents constant total scores of 0, both for trabecular bone and articular cartilage, with a mean of 0 and standard deviation of 0. These results confirm the complete absence of morphological alterations, indicating a healthy and homogeneous tissue structure.

The pathological group, on the other hand, has total scores ranging from 5 to 8, with a mean of 6.14 and a standard deviation of approximately 1.1. All three criteria—structural continuity, collagen organization, and vascularization—indicate marked impairment:

- Structural continuity: mean scores are 2.0–2.5, indicating a significant loss of tissue integrity;
- Fibrillar organization: mean scores are 2.5–3.0, reflecting severe collagen disorganization;
- Vascularization: mean scores are 2.0–2.5, with evidence of abnormal vascular networks;
- Comparison of the mean SEM total scores between the pathological group (6.14) and the control (0) reveals a clear and significant difference (*p* < 0.001).

These findings demonstrate that morphological changes are an objective indicator of degenerative disease progression.

#### 3.4.3. Correlation of the Statistical Results

The results obtained by scanning electron microscopy (SEM) for the group of patients (51 cases) highlight the following aspects:

- Continuity/structural integrity: total score of 102, with an average of 2.0 per patient, which indicates a moderate impairment of the tissue structure;
- Presence of collagen/fibrillar organization: total score of 124, with an average of 2.43, which suggests a significant disorganization of the collagen network;
- Vascularization/visible capillaries: total score of 102, with an average of 2.0 per patient, which reflects the abnormal presence of blood vessels in the analyzed cartilage.

These indicators highlight marked morphological alterations that are specific to degenerative joint processes.

From a statistical point of view, the group of patients presents significantly increased SEM scores, with averages between 2.0 and 2.43, which indicates a constant and pronounced impairment of the tissue structure. This distribution of values highlights a systematic morphological degradation that is specific to degenerative joint pathologies. The reduced structural integrity, disorganized collagen, and abnormal vascularization suggest a chronic inflammatory process, which is probably supported by high IHC scores. When compared to the control group, the differences are statistically significant, reflecting an advanced stage of the disease, both morphologically and biochemically.

Within the patient group, a clear trend of positive association is observed between high SEM scores and increased IHC scores. Thus, patients presenting with significant morphological impairment—characterized by structural discontinuities, collagen fibrillar disorganization, and abnormal vascularization—tend to have high IHC scores, especially for markers involved in inflammation (IL-1β, TNF-α), enzymatic degradation (MMP-13, ADAMTS), and tissue remodeling (TGF-β, VEGF—often associated with excessive vascularization).

## 4. Discussion

The femoral head contains a complex vascular network composed of central and synovial vessels that are essential for bone and cartilage health. In this study, comparative analysis between patients with degenerative hip pathology and healthy controls revealed significant differences in both immunohistochemical (IHC) profiles and ultrastructural features, as assessed via scanning electron microscopy (SEM).

SEM imaging of degenerative samples showed architectural disorganization, disrupted collagen matrix, and diminished microvascular networks, whereas control specimens preserved a well-organized lamellar bone structure with intact vasculature. These structural changes correlated with the IHC findings. The vascular integrity was assessed with CD31 and ERG. Controls showed strong, continuous staining with both markers, corresponding to preserved capillary networks observed in SEM. In contrast, degenerative tissues showed reduced and irregular CD31 and ERG expression, which is consistent with disrupted vessel morphology and ischemic zones. CD31 serves as a key endothelial marker of vascular integrity [13], and its attenuation in degenerative samples reflects microvascular compromise [14,15,16,17].

Ki67, a marker of proliferation, was significantly elevated in degenerative samples, which indicates increased but disordered cell turnover, consistent with chaotic tissue remodeling [18].

Inflammation was evaluated using CD68, a macrophage marker. Degenerative tissues showed moderate CD68 positivity, reflecting chronic low-grade inflammation and correlating with SEM-detected resorption lacunae. Controls exhibited minimal CD68 expression, which suggests quiescent inflammatory status. This observation aligns with current hypotheses that propose that chronic macrophage activity modulates cartilage and bone degeneration in osteoarthritis and osteonecrosis [19,20].

The SOX9 expression remained relatively unchanged between groups, which implies that basal chondrogenic transcription persists. However, the SEM findings revealed matrix fragmentation and loss of the supportive environment required for regeneration, indicating a disconnect between molecular potential and structural function [21,22].

Lubricin, a critical surface glycoprotein, was significantly reduced in degenerative samples. SEM corroborated this through surface fibrillation and erosion, indicating compromised lubrication. Lubricin reduction is known to precede structural degradation and may serve as an early biomarker of cartilage pathology [23,24,25].

Together, these findings support the concept that degenerative hip disease involves a cascade of molecular alterations—elevated Ki67, chronic CD68-mediated inflammation, impaired vascularization (CD31, ERG), and early loss of lubricin—accompanied by structural breakdowns observed via SEM. In contrast, control samples exhibited cohesive IHC and SEM profiles: intact vascularization, low proliferative and inflammatory indices, and preserved matrix architecture.

Quantitative comparisons supported these observations. Pathological samples showed increased IHC scores for Ki67 (2.21 vs. 1.80, *p* = 0.002) and ERG (2.25 vs. 1.80, *p* = 0.009), and significantly reduced lubricin (0.78 vs. 2.33, *p* < 10^−8^). Interestingly, the CD68 scores were slightly lower in the degenerative group (0.16 vs. 0.60, *p* = 0.005), possibly reflecting chronic, non-acute inflammation. No significant differences were found for CD31 (*p* = 0.58) or SOX9 (*p* = 0.94), which aligns with previous findings [13,14,15,16,21,22].

SEM–IHC correlation analysis confirmed that, in the pathological samples, higher SEM scores (≥6) were associated with elevated IHC scores (2–3), especially in areas showing vascular SEM scores of 2–3 and correspondingly high VEGF expression. This reinforces the link between molecular dysregulation and structural degradation. In contrast, control samples had uniformly low SEM and IHC scores (0–1), demonstrating structural integrity and minimal degeneration.

Further SEM evaluation revealed distinct vascular patterns: degenerative samples showed fragmented trabeculae, perforations, and loss of periendothelial coverage in remaining vessels, which point to impaired angiogenesis. Previous studies support that disrupted vascular supply—particularly arteriosclerosis—impairs bone quality and healing capacity [14,17]. Experimental porcine models have been used to map the femoral head vasculature, offering insight into osteonecrosis and osteoporosis [14,26,27].

Chondrocyte proliferation, observed via Ki67, is often a compensatory early event that is followed by apoptosis in late-stage degeneration [1,2]. This aligns with the detection of chondrocyte lacunae collapse by SEM. Additionally, chronic macrophage presence (CD68+) interferes with regeneration, reinforcing the inflammatory component in osteoarthritis and osteonecrosis [19,28].

ERG and CD31 jointly allow precise vascular profiling, which is essential in evaluating angiogenic responses [29,30,31,32]. Reduced expression signals failed neovascularization and disease progression. The persistence of SOX9 without matrix regeneration may indicate latent chondrodysplasia shifts [24,33,34].

This integrative approach, correlating molecular expression with ultrastructural morphology, surpasses prior single-marker studies, offering comprehensive diagnostic and prognostic insights [25,35,36]. The absence of a gold-standard protocol for early joint degeneration highlights the value of this combined SEM–IHC methodology in diagnosis and staging [2].

An additional limitation of the present study is the lack of standardized bone mineral density (BMD) data across all included patients. Although the BMD may influence the subchondral architecture and trabecular remodeling, its inconsistent availability precluded meaningful integration into our analysis. Future studies could benefit from incorporating DXA-based BMD assessments to further explore correlations between mineral density and vascular or ultrastructural changes in degenerative joint disease.

Statistical analysis confirmed group-specific patterns, with consistent SEM–IHC correlations being observed only in degenerative tissues (ρ > 0.6, *p* < 0.05), indicating a reliable biomarker relationship under pathological conditions. No sex-based differences were found.

In our study, degenerative joint disease is characterized by parallel structural and biochemical disruptions. The coordinated use of Ki67, CD68, CD31, ERG, SOX9, and lubricin, together with SEM imaging, provides a multidimensional framework for understanding tissue degeneration.

## 5. Conclusions

The integration of SEM and IHC findings thus offers a powerful, multidimensional perspective on the degenerative cascade, emphasizing the critical interdependence between molecular alterations and microarchitectural deterioration in the pathogenesis of hip joint diseases. These findings highlight that degenerative hip disease is not merely the result of mechanical wear, but rather a complex biological process in which cellular proliferation, chronic inflammation, vascular impairment, and extracellular matrix degradation act synergistically to compromise tissue integrity. While decreased vascularity might be expected to limit cellular activity, previous research has shown that local hypoxic conditions within degenerative bone and cartilage can paradoxically trigger pro-inflammatory signaling cascades and promote compensatory cellular proliferation. Hypoxia-inducible factors (HIFs), in particular, are known to be upregulated in osteoarthritic joints and can stimulate inflammatory cytokine expression and matrix remodeling, even in the context of reduced perfusion.

Moreover, the proliferation observed in our specimens is likely localized and patchy, corresponding to regions of reactive remodeling rather than diffuse hyperplasia. These focal proliferative responses may represent an attempted repair mechanism rather than a sustained regenerative process. This is the etiopathogenic hypothesis that we arrived at as a result of our study and which needs to be confirmed by future research.

The combined use of scanning electron microscopy and immunohistochemical profiling thus emerges as a robust diagnostic strategy, offering comprehensive insights into both the structural and molecular underpinnings of joint degeneration. Future therapeutic approaches should consider targeting these interconnected pathways to more effectively prevent or slow the progression of degenerative hip pathology.

However, this study faced several challenges, particularly in preserving tissue integrity during the sample preparation for SEM analysis and in ensuring optimal antigenicity for IHC evaluation across highly mineralized bone structures. The complexity of correlating ultrastructural and molecular data also required meticulous matching of anatomical regions and rigorous standardization of imaging and staining protocols.

Despite these limitations, this study opens promising avenues for future research. The detailed correlation between microarchitectural disruption and specific molecular alterations paves the way for the development of integrated diagnostic models. Such models could enable earlier detection of degenerative changes, stratification of disease severity, and identification of novel therapeutic targets aimed at preserving vascular health, modulating inflammation, and maintaining extracellular matrix integrity in degenerative joint diseases.

Future therapeutic strategies should focus on preserving vascular health, modulating chronic inflammation, and supporting matrix regeneration to manage or prevent disease progression.

## Figures and Tables

**Table 1:** The table presents the immunohistochemical (IHC) reaction intensity scores obtained for 66 patients, divided into the two study groups; the tissue analyzed for scoring is articular cartilage, and the scores were estimated semi-automatically (ImageJ open source), based on digital analysis of IHC staining intensity.

Patient ID	Lot	Ki67	CD68	CD31	SOX9	ERG	Lubricin
1	Pathologic	2	0	2	3	3	1
2	Pathologic	3	0	2	3	2	1
3	Pathologic	2	0	2	3	2	2
4	Pathologic	3	0	3	3	3	1
5	Pathologic	2	0	2	2	3	0
6	Pathologic	2	1	2	3	2	0
7	Pathologic	2	0	2	3	2	1
8	Pathologic	3	0	2	3	2	0
9	Pathologic	2	0	2	3	2	1
10	Pathologic	2	0	2	2	2	1
11	Pathologic	2	0	2	2	2	0
12	Pathologic	2	1	3	3	2	0
13	Pathologic	3	0	2	3	2	1
14	Pathologic	2	0	2	3	3	2
15	Pathologic	2	0	2	3	2	1
16	Pathologic	2	0	2	3	2	1
17	Pathologic	2	0	2	2	2	1
18	Pathologic	2	0	2	3	3	1
19	Pathologic	3	1	3	3	3	1
20	Pathologic	2	0	2	3	3	1
21	Pathologic	2	0	2	2	2	0
22	Pathologic	2	0	2	3	2	1
23	Pathologic	2	0	2	3	2	1
24	Pathologic	2	1	3	3	2	1
25	Pathologic	2	0	2	3	2	0
26	Pathologic	3	0	2	2	2	0
27	Pathologic	2	0	2	3	2	0
28	Pathologic	2	0	3	3	2	0
29	Pathologic	3	0	2	3	2	1
30	Pathologic	2	0	2	3	3	1
31	Pathologic	2	0	2	3	2	1
32	Pathologic	2	0	2	2	2	1
33	Pathologic	2	0	2	3	2	1
34	Pathologic	2	1	3	3	2	1
35	Pathologic	2	0	2	3	2	2
36	Pathologic	3	0	3	3	3	1
37	Pathologic	2	1	2	2	2	1
38	Pathologic	2	0	2	3	2	0
39	Pathologic	3	0	2	3	2	0
40	Pathologic	2	0	2	2	3	0
41	Pathologic	2	0	2	3	3	2
42	Pathologic	2	0	3	2	3	1
43	Pathologic	3	0	2	2	3	2
44	Pathologic	2	0	2	3	2	2
45	Pathologic	2	0	2	3	2	1
46	Pathologic	2	0	1	3	2	1
47	Pathologic	2	0	2	3	2	1
48	Pathologic	2	1	2	3	2	0
49	Pathologic	2	0	3	2	2	0
50	Pathologic	3	0	3	2	2	0
51	Pathologic	2	0	2	3	3	1
52	Control	2	1	2	3	2	2
53	Control	2	1	2	3	2	2
54	Control	2	1	2	3	2	3
55	Control	2	0	1	2	3	3
56	Control	2	1	1	3	2	3
57	Control	1	1	2	3	2	3
58	Control	2	0	2	1	1	2
59	Control	2	0	3	3	1	2
60	Control	2	1	3	3	2	2
61	Control	1	0	3	3	3	2
62	Control	2	1	2	2	2	3
63	Control	2	0	2	3	3	3
64	Control	2	1	2	3	1	3
65	Control	1	0	2	3	2	2
66	Control	2	1	2	2	2	2

**Table 2:** SEM morphological scoring—trabecular bone and articular cartilage (control vs. pathological group).

SEM Figure	Lot	Analyzed Structure	Continuity/Structural Integrity	Collagen Presence/Fibrillar Organization	Visible Vascularization/Capillaries	Total Score (0–9)
III.3.1.1	Control	Trabecular bone	0	0	0	0
III.3.1.2	Control	Articular cartilage	0	0	-	0
III.3.2.1	Pathologic	Trabecular bone	3	3	2	8
III.3.2.2	Pathologic	Trabecular bone	2	2	2	6
III.3.2.3	Pathologic	Epiphyseal vascularization	2	2	3	7
III.3.2.4	Pathologic	Haversian vascularization	1	2	2	5
III.3.2.5	Pathologic	Collagen matrix	2	3	1	6
III.3.2.6	Pathologic	Trabecular vascularization	1	2	2	5
III.3.2.7	Pathologic	Articular cartilage	3	3	-	6

**Table 3:** The table represents the cumulative scores obtained in the morphological analysis by scanning electron microscopy (SEM) of bone and cartilage samples from patients with degenerative pathology of the hip joint and from subjects with normal joint (control group).

Lot	Number of Cases	Total Continuity/Structural Integrity Score	Total Score Collagen Presence/Fibrillar Organization	Total Score Vascularization/Visible Capillaries
Control	15	0	0	0
Pathologic	51	102	124	102

## Data Availability

Data are contained within the article.
